# GIN‐McMaster Guideline Development Checklist Extension for Integrating Artificial Intelligence in the Health Guideline Enterprise

**DOI:** 10.1002/gin2.70073

**Published:** 2026-05-30

**Authors:** Manuel Marques‐Cruz, Bernardo Sousa‐Pinto, Ignacio Neumann, Yuan Chi, Monika Nothacker, Artur Nowak, Marge Reinap, Mariette Awad, Pablo Alonso‐Coello, Amir Qaseem, Elie A. Akl, Wojtek Wiercioch, Jan Brozek, Holger J. Schünemann, the GIN AI working group

**Affiliations:** ^1^ MEDCIDS ‐ Department of Community Medicine, Information and Health Decision Sciences; Faculty of Medicine University of Porto Porto Portugal; ^2^ CINTESIS@RISE ‐ Health Research Network, MEDCIDS, Faculty of Medicine University of Porto Porto Portugal; ^3^ School of Medicine Universidad San Sebastián Santiago Chile; ^4^ Clinical Epidemiology and Research Center (CERC) Humanitas University Milan Italy; ^5^ Yealth Network, Beijing Yealth Technology Co., Ltd Beijing China; ^6^ AWMF (Association of the Scientific Medical Societies in Germany) Institute for Medical Knowledge Management, c/o University of Marburg, Marburg Germany; ^7^ Evidence Prime Hamilton Ontario Canada; ^8^ WHO Regional Office for Europe Copenhagen Denmark; ^9^ Department of Electrical and Computer Engineering American University of Beirut Beirut Lebanon; ^10^ Institut de Recerca Sant Pau (IR Sant Pau), and CIBER Epidemiología y Salud Pública (CIBERESP) Barcelona Spain; ^11^ American College of Physicians Pennsylvania Philadelphia USA; ^12^ Department of Internal Medicine American University of Beirut Beirut Lebanon; ^13^ Department of Health Research Methods, Evidence and Impact, and the Department of Medicine McMaster University Hamilton Ontario Canada; ^14^ Michael G. DeGroote Cochrane Canada & McMaster GRADE Centres, McMaster University Hamilton Ontario Canada; ^15^ Allergy and Immunology Charité, Universitätsmedizin‐Berlin Berlin Germany

## Abstract

**Introduction:**

Artificial intelligence (AI) may support several processes of the health guideline enterprise. This article describes the development of an extension of the Guidelines International Journal (GIN)‐McMaster Guideline Development Checklist (GDC) for integrating AI in the guideline enterprise. This development has been led by the GIN‐AI Working Group.

**Methods:**

We started by prompting a large language model (LLM) for items related to the use of AI in each of the steps of the original GDC. Subsequently, the members of the working group engaged in a set of iterative discussions, resulting in item refinement and in a consensus first version of the extension. We retrospectively applied this first version to a case use of guidelines incorporating AI in their development (Allergic Rhinitis and its Impact on Asthma [ARIA] 2024‐2025 guidelines), leading to further refinement and to the approval of the final extension tool.

**Results:**

Prompting LLMs resulted in the generation of 149 items. Of those, 117 were removed and 19 were modified by members of the working group. On the other hand, 17 new items were added during the iterative discussion process. The retrospective application of the extension led to changes in the wording of four items. The final version of the checklist extension has been approved with 49 items modifying or adding to the original GDC.

**Discussion:**

We have developed an extension of the GIN‐McMaster GDC that encompasses a set of conduct standards that are intended to facilitate the comprehensive and transparent integration of AI in the health guideline enterprise.

**Clinical Trial Registration:**

Not applicable. This study is not a clinical trial.

## Summary

The GIN‐McMaster Checklist for Integrating Artificial Intelligence (AI) in the Health Guideline Enterprise provides guidance and practical tips for how to consider AI in guideline planning, development and implementation.

## 1 Introduction

Even though there is no universal definition of artificial intelligence (AI), it can be broadly understood as “systems that display intelligent behaviour by analysing their environment and taking actions—with some degree of autonomy—to achieve specific goals” [1]. This “degree of autonomy” distinguishes AI from algorithms based on deterministic automation rules, resulting in new opportunities and challenges for its use in daily practice (e.g., hallucinations are not expected with deterministic algorithms). Of note, this article focuses on the health guideline enterprise rather than AI itself, so we will intentionally simplify AI‐related topics without attempting to be comprehensive or technically accurate from a computer science perspective.

As with other fields, there has been a growing interest on the use of AI in the evidence cycle. Examples of such use include study selection and data extraction in systematic reviews [2, 3]. It is expected that the use of AI will also become more common in the guideline enterprise. In this context, considering that the use of AI is not devoid of risks, the Guidelines International Network (GIN) has created a working group on AI. Its first tasks were (i) the identification of principles for the development and use of AI tools to support the health guideline enterprise [4], and (ii) the creation of an extension to the GIN‐McMaster Guideline Development Checklist (GDC) for the use of AI in guidelines. These two tasks are complementary and inter‐related. The implementation of the principles for the use of AI in the guideline enterprise can be supported by using this extension of the GIN‐McMaster GDC.

In this article, we provide the extension of the GIN‐McMaster GDC for integrating AI in the guideline enterprise and describe its development (with a particular focus on generative AI, such as large language models [LLMs]). The GIN‐McMaster GDC is a de facto standard for considering all aspects in guideline development [5]. Several extensions to the GIN‐McMaster GDC have been published, including those for rapid guidelines [6], interest‐holder involvement [7], considering equity [8], and for the integration of quality improvement and quality assurance [9]. The extension we are proposing adds items to the GIN‐McMaster GDC dealing with the use of AI at the different stages of the guideline enterprise.

## 2 Methods

We provide a complete description of the methods used to develop this checklist extension in the published protocol [10]. The protocol was followed except for the fact that, due to time constraints, we were not able to do a prospective user testing of the checklist extension. Instead, we have retrospectively applied it to existing guidelines.

We established a GIN working group on the role of using AI in the context of health guidelines to address its opportunities, risks, and limitations. The working group started with 14 guideline developers (including three members of the GIN board) from most regions of the world with backgrounds in evidence‐based medicine, guideline development, data science, and AI (Supplementary Table 1).

### 2.1 Study Design

We followed a multi‐phase approach to generate a list of items concerning the integration of AI tools in guideline development based on the original GDC and its 18 steps. We conducted a scoping review on the use of AI in health guidelines that was used to support the decisions during the development of the checklist extension. We prompted ChatGPT 4.0 in February 2024 for relevant items on the use of AI tools for each guideline development step. Members of the working group reviewed and revised the outputs in two stages: 1) iterative refinement performed independently by two members of the working group, and 2) internal evaluation through consensus by all working group members. We tested the resulting proposal for the checklist extension by retrospective application to the Allergic Rhinitis and its Impact on Asthma (ARIA) 2024‐2025 guidelines [11].

### 2.2 Preparatory Phase: Scoping Review

We performed a scoping review searching MEDLINE and Embase for articles about the use of AI tools in the guideline enterprise. We described the methods of the scoping review and its results elsewhere [4]. In the specific context of the development of the GIN‐McMaster GDC extension, the aim of the scoping review was to identify any existing checklist for the use of AI in the guideline enterprise, support members of the working group in the revision of the output obtained from ChatGPT 4.0, and prepare group members for phases 2 and 3 of the checklist extension development.

### 2.3 Phase 1: AI‐Assisted Item Generation

As a starting point for the development of the checklist extension, we prompted ChatGPT 4.0 for a list of relevant items related to the use of AI tools in each of the 18 guideline development steps of the original GDC [5]. We provided as context the items from the GDC (topic labelled as a “General/Non‐specific”) and the items from the GIN‐McMaster Checklist Extension for Quality Assurance and Quality Improvement [9] (topic labelled as “Integrating Quality Assurance and Improvement in guideline development”). Box 1 includes the original prompt. When prompting ChatGPT 4.0 we followed the GIN principles for the use of AI in the health guideline enterprise [4]. We followed a few‐shot approach in which the items provided as context were used to define the expected structure and format of the model's outputs. Specifically, the topic and guideline development step were provided as user input to frame the task, and the corresponding items were supplied as assistant messages, as if they were model‐generated answers. This resulted in a “System–User–Assistant–User–Assistant–User” prompt structure. The final user message then required the model to complete the subsequent assistant turn. In this way, the prompt demonstrated the task and output format through examples, while the final assistant response constituted the model's generated result.

Box 1 **Prompt structure for obtaining guideline extension items with ChatGPT 4.0.**

**System:** You are an helpful assistant. Your role is to propose all relevant topic‐specific guideline development items to a list of pre‐specified steps. A user will provide a topic and a guideline development step. You will answer with the items, separated by ‘;’ when more than one, or with ‘None’ if no additional items are relevant.

**User:** Topic ‐ General/Non‐specific; Guideline development step ‐ [*one of the 18 development steps*]

**Assistant:** [*all items in the GDC for the specified development step*]

**User:** Topic ‐ Integrating Quality Assurance and Improvement in guideline development; Guideline development step ‐ [*one of the 18 development steps*]

**Assistant:** [*all items in the GDC for the specified development step*]

**User:** Topic ‐ Integrating Artificial Intelligence tools in guideline development; Guideline development step ‐ [*one of the 18 development steps*]

### 2.4 Phase 2: Initial Iterative Refinement

Two members of the GIN working group on AI (MMC and BSP) independently assessed the items resulting from prompting ChatGPT 4.0. Such an assessment involved (i) removal of duplicate items, (ii) comparison of the proposed items with the original GDC checklist and the Quality Assurance and Quality Improvement extension, (iii) deletion of the items which did not refer to the use of AI tools for guideline development, and (iv) addition of items found in other relevant checklists identified in the scoping review. After each of the two working group members completed their independent assessment, a discussion was held to reach consensus between them and identify any remaining items to be added or existing items that would require further deletions or modifications.

### 2.5 Phase 3: Internal Working Group Consensus

All working group members provided feedback on the draft list of items using an online form asking each member (i) to decide on the inclusion or exclusion of each proposed checklist item, (ii) to propose modifications to the existing items, and (iii) to suggest relevant items to add. After receiving the feedback we edited the list and we iteratively sent the form to the group members until no new suggestions were provided. Were drafted the checklist. extension and held a meeting of all working group members to discuss the draft and approve the checklist extension proposal by consensus.

### 2.6 Phase 4: Application to a Sample Guideline

Considering that the prospective piloting of the checklist in a guideline would require a long period of time, we tested it by retrospectively applying it to the ARIA 2024–2025 guidelines. The ARIA 2024–2025 guidelines concern the treatment of allergic rhinitis [11]. We selected the ARIA guidelines to test the checklist extension because AI tools were used during its development for question and outcome generation, and to support the evidence search. Of note, at the time of the user testing, the ARIA 2024–2025 had not yet been fully completed: two of their reports (focused on nasal treatments and oral treatments) had been drafted but not yet published, so that dissemination and implementation had not yet occurred.

A member of the ARIA 2024–2025 guidelines steering group assessed the guidelines using the checklist extension, classifying each item as either fulfilled, not fulfilled or not applicable, providing a brief justification for each decision and recording any suggestions for item modification.

### 2.7 Phase 5: Final Approval

All working group members provided feedback on the suggested modifications after the testing of the checklist extension. We purposely built an online form asking each member to indicate agreement or disagreement with each suggested change. We held a meeting to discuss and approve the final version of the checklist extension. We then submitted the checklist to the GIN board for review.

## 3 Results

**Figure 1:**
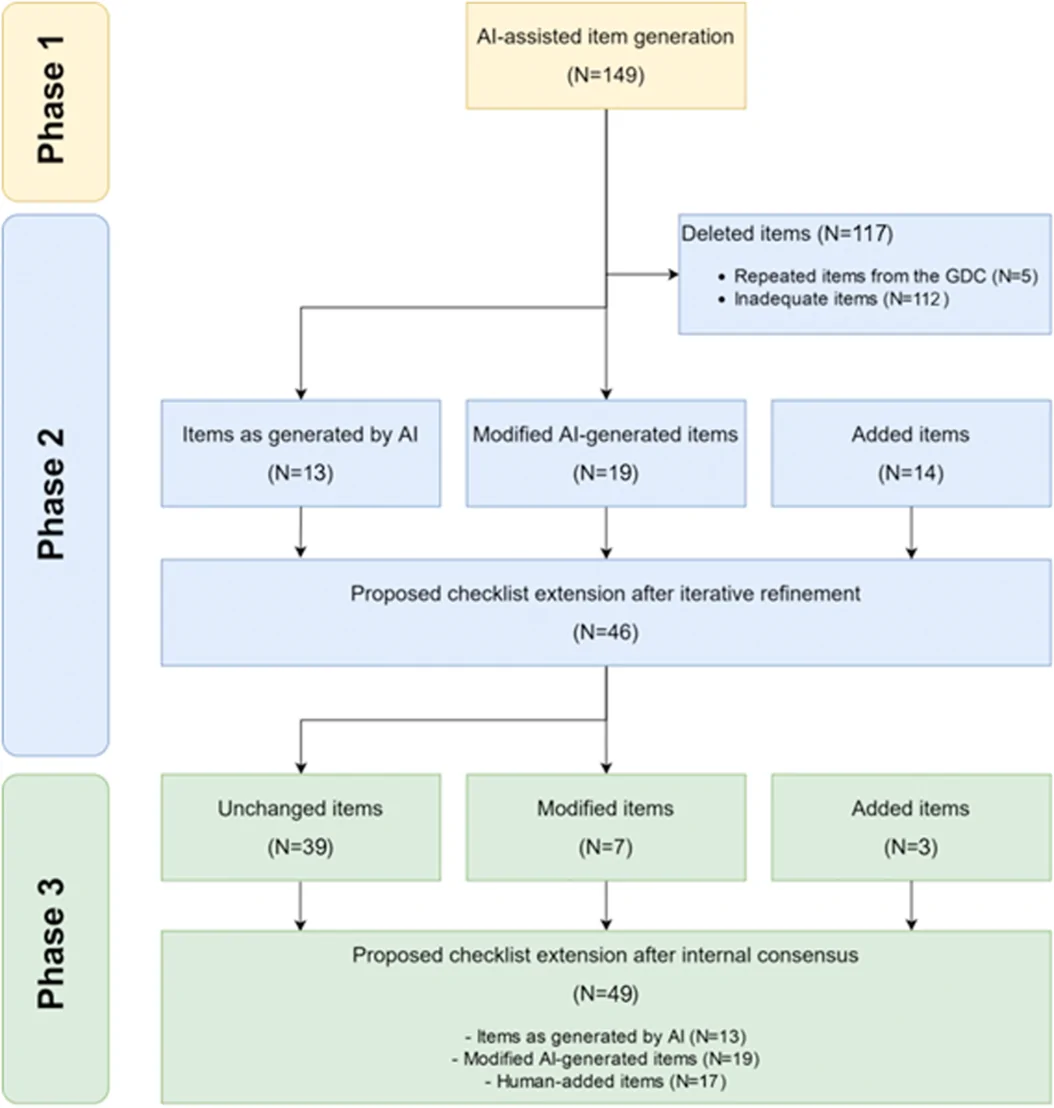
Flow diagram of item generation and refinement for integration of artificial intelligence (AI) tools in guideline development checklist (GDC) extension.

The flow diagram in Figure 1 provides a description of the number of items added, modified or excluded in each phase. AI‐assisted item generation produced 149 items. We removed 117 items (79.2%) because they were duplicates of items already included in the GDC (*N* = 5) or they were not related to the use of AI tools for guideline development (*N* = 112) (Table 1). We also modified 19 of the remaining 32 AI‐generated items (59.4%) and added 14 new items. Therefore, only 8.7% from the items generated by ChatGPT 4.0 remained in the checklist extension proposal without modification other than minor editorial changes. Of note, none of the 14 added items was part of other published checklists, as no other checklist on AI for the guideline enterprise was found in the scoping review.

**Table 1:** Summary of the number of items resulting from ChatGPT 4.0 prompting and iterative refinement.

Topic	Proposed items (ChatGPT 4.0)	Repeated from GDC	Deleted (total)^a^	Modified	Added
1. Organization Budget Planning and Training	7	0	2	1	1
2. Priority Setting	8	2	7	1	1
3. Guideline Group Membership	7	1	4	1	0
4. Establishing Guideline Group Processes	9	0	4	2	0
5. Identifying Target Audience and Topic Selection	5	0	5	0	0
6. Document the processes	9	0	9	0	0
7. Conflict of Interest (COI) Considerations	8	0	3	1	1
8. (PICO) Question Generation	9	1	8	1	1
9. Considering Importance of Outcomes and Interventions	9	0	8	1	4
10. Deciding what Evidence to Include and Searching for Evidence	9	0	7	2	0
11. Summarizing Evidence and Considering Additional Information	9	0	9	0	1
12. Judging Quality Strength or Certainty of a Body of Evidence	6	1	5	1	1
13. Developing Recommendations and Determining their Strength	10	0	9	1	1
14. Wording of Recommendations and Considerations	10	0	9	1	1
15. Reporting and Peer Review	9	0	7	2	0
16. Dissemination and Implementation	9	0	7	2	1
17. Evaluation and Use	8	0	7	0	1
18. Updating	8	0	7	1	0
Total	149	5	117	18	14

^a^ includes both repeated items from GDC without reference to the use of AI tools in guideline development and other inadequate items.

The first draft of the checklist extension comprised of 46 items (Supplementary Table 2). In the iterative consensus phase, we further modified seven items and added three new items. The revised draft checklist extension after internal working group consensus included 49 items (Table 2) of which 13 (26.5%) remained as originally generated, 19 (38.8%) corresponded to modified AI‐generated items, and 17 (34.7%) to human‐suggested items.

**Table 2:** Checklist extension items resulting from ChatGPT 4.0 prompting, iterative refinement, internal consensus, and application to a sample guideline.

Topic	Additional Items for AI tools integration in guideline development (items that should be in addition or integrated with original checklist items)
1. Organization budget planning and training	1. Identify the specific AI tools that could be integrated into specific stages of the guideline development process, such as evidence synthesis, data analysis, guideline dissemination, and implementation tracking. 2. Assess the technical and time requirements and infrastructure needed to support the integration of AI tools in the guideline development process, including software, hardware, and technical expertise. 3. Develop a framework to ensure that selected AI‐tools will remain current throughout the guideline development process. 4. *Correspondence with item 4 of the original GDC:* Evaluate the costs associated with integrating AI tools into the guideline development process, including software licenses, training for team members, and potential ongoing support and maintenance fees. 5. Identify and establish partnerships with AI technology providers or academic institutions with expertise in AI to support the optimisation* and/or integration of these tools into the guideline development process. 6. *Correspondence with item 8 of the original GDC:* Plan for training sessions for guideline development team members on the use of AI tools, ensuring they understand how to effectively use these technologies in their work and that they understand their limitations. 7. Get eventual needed approvals from advisory and/or regulatory bodies for the guideline development process.
2. Priority setting	1. Develop or apply a framework for using AI to support the priority setting process and guideline topic selection, for example to aid in the collection of evidence to inform priorities (e.g., assessing topic impact, identifying recent research evidence, assessing the availability and credibility of other recent guidelines, or identifying implementation issues and barriers to change) (*see also items 5 and 7 of the original GDC*). 2. *Modification to item 9 of the original GDC:* Document the priority setting process, including how AI tools were used.
3. Guideline group membership	1. Include AI and data science experts in the guideline development group to provide expertise on the integration, application and limitations of AI tools within the guideline development process. 2. Consider the need for interdisciplinary collaboration, involving software developers, data scientists/analysts, among others, to develop/optimise, integrate and use AI tools effectively. 3. Establish roles for AI experts within the group, such as advising on AI tool selection, overseeing the development of custom AI solutions, ensuring the quality and reliability of AI applications, and explaining AI‐generated outputs and their limitations.
4. Establishing guideline group processes	1. Establish a workflow for how AI‐generated evidence, insights and related outputs will be incorporated into the guideline development process, ensuring transparency and accountability in how AI outputs are used. 2. *Modification of item 3 of the original GDC:* As part of the training for the guideline development group, ensure that group members understand what the process and proposed methods will be and that they need to be adhered to (e.g. consensus methods that may be used, anonymous or non‐anonymous voting, assessment of evidence and of AI‐generated outputs, understanding of AI methodologies relevant to the tasks, group discussion and contributing ideas). 3. Establish communication channels for collaboration between AI experts and other members of the guideline development group, to facilitate knowledge exchange and integration of AI into the guideline process. 4. *Adding to item 5 of the original GDC:* Determine how disagreements regarding AI‐based outputs or their interpretations will be resolved, ensuring a fair and evidence‐based approach. 5. Implement feedback mechanisms to evaluate and continuously improve the impact of AI tools on the guideline development process, ensuring ongoing transparency and the ability to adapt to technological advances.
5. Identifying target audience and topic selection	None.
6. Consumer and stakeholder involvement	1. Consider the use of AI tools to analyse or summarise the inputs of different stakeholders.
7. Conflict of interest (COI) considerations	1. Extend the declaration of interest policy to include potential conflicts specific to AI tool development, deployment, and evaluation, such as financial interests in AI technologies that can be used in guideline development. 2. Require disclosure of any intellectual property interests in AI technologies that could influence recommendations or the selection of AI tools for guideline development. 3. Implement a policy for managing COI related to AI, such as excluding individuals with significant conflicts from making decisions on the selection of AI tools, while allowing for their contribution in areas where their expertise is essential but their COI does not influence outcomes. 4. Disclose and publish any conflicts of interest related to AI technologies among guideline group members, including the nature of the conflict and how it was managed, to maintain transparency and trust in the guideline development process. 5. Regularly update COI declarations to reflect any new AI‐related interests or partnerships that could affect the integrity of the guideline development process. 6. Explicitly and transparently disclose whether AI tools were employed for supporting the assessment or management of conflicts of interest, and detail their specific applications.
8. (PICO) question generation	1. Detail how AI tools were applied for supporting the identification of PICO questions based on infodemiology data or other sources of data. [12] 2. Detail how AI tools were applied for directly generating PICO questions, including prompts used, the roles the AI tools were asked to assume, and whether there were was an evaluation of the potential impact of AI biases in the direct generation of questions.
9. Considering importance of outcomes and interventions	1. Consider, plan and detail the application of AI tools for supporting the identification of health outcomes based on infodemiology data or data in natural language from other sources. 2. Consider, plan and detail the application of AI tools for direct identification of potentially relevant outcomes, including prompts used and the roles the AI tools were asked to assume. 3. Describe the AI‐assisted outputs in processes of evidence collection, analysis, and synthesis for systematic reviews of patients' values. [13] 4. Indicate whether AI tools were used for the appraisal of the risk of bias and certainty of the evidence in the evaluation of patients' values. 5. Thoroughly and transparently record the processes involving the use of AI tools for eliciting values and preferences or health utilities, including considerations about the risk of algorithm biases resulting from the use of AI tools (e.g., risk of underrepresentation of the perspectives of some patient groups or populations).
10. Deciding what evidence to include and searching for evidence	1. Establish a process for engaging with AI developers and researchers to select and use AI tools for evidence search, analysis, and/or synthesis in systematic reviews that will be conducted. 2. Document all the processes in which AI tools were used (e.g., record selection, data extraction, risk of bias assessment, identification of unpublished data) to obtain, analyse and synthesise evidence required for the guideline, ensuring transparency and—as much as possible ‐ reproducibility.
11. Summarizing evidence and considering additional information	1. Document how AI tools were used to extract data and summarize evidence including based on information from different sources (e.g., systematic reviews, collection of primary data, etc.). Detail the human validation process for AI‐extracted data and summaries, including error rates or agreement metrics if assessed.
12. Judging quality strength or certainty of a body of evidence	1. *Modification of item 2 of the original GDC:* Decide who will be responsible for appraising the quality of evidence (e.g. un‐conflicted methodologists participating in the working group) and whether AI tools will be used as a support. 2. Document how AI tools were used for evaluating the certainty in the body of evidence, clearly indicating the domains which were and were not assessed with AI assistance, and how AI‐based classifications were validated. 3. Ensure the consistency of AI tool performance in the assessment of the certainty of evidence across different outcomes and comparisons in the guideline.
13. Developing recommendations and determining their strength	1. Document the methods by which AI tools were used to support the development of recommendations, clearly delineating between AI and human roles in the development of recommendations. 2. Define methods for assessing the quality and credibility of AI outputs for supporting judgements and recommendations in the context of the Evidence‐to‐Decision framework.
14. Wording of recommendations and considerations of implementation, feasibility and equity	1. Document the use of AI tools in the drafting of recommendation wording (e.g., generative wording in a standardised format with precise and unambiguous language). 2. Document the use of AI tools for adaptation of recommendation wording to different stakeholders, including whether role‐playing scenarios were employed to tailor recommendations for various stakeholders (e.g., patients of different backgrounds, healthcare providers, policymakers).
15. Reporting and peer review	1. Ensure the guideline report explicitly addresses how AI tools were used for guideline development, specifying the tools and methodologies employed. 2. Conduct a thorough review of the AI‐specific outputs in the guideline by experts in AI, data science, and healthcare to ensure accuracy, relevance, and completeness.
16. Dissemination and implementation	1. Create a detailed workflow that identifies areas where AI tools can enhance comprehensive dissemination strategies. This should include targeted and customized communication approaches for healthcare professionals, technology developers, policymakers, patients and the public. 2. Document how AI tools were used for creation of dissemination and implementation products (e.g., writing of plain language summaries, design of decision‐aids and guideline summaries). 3. Detail how AI tools were used for the identification and support of the overcome of obstacles and challenges to implementation of guideline recommendations. 4. Partner with healthcare technology organizations and AI developers to facilitate the integration of guideline recommendations into AI tools (e.g., AI‐based clinical decision support systems or interactive bots), ensuring alignment with best practices.
17. Evaluation and use	1. Provide guidance on the criteria for modifying, continuing, or discontinuing the use of specific AI tools in the guideline development process based on evidence from evaluations and changing technological landscapes. For example, after guideline completion, evaluate the efficiency, cost‐effectiveness and quality impact of using AI tools in the development process. 2. Detail if AI tools will be used to monitor and audit the implementation and effectiveness of guideline recommendations, including specific outcomes to track and methods for their assessment.
18. Updating	1. Secure resources and plan logistics for the efficient update of the guideline, considering the need for ongoing collaboration with AI developers and researchers to ensure that AI tools can be leveraged for identifying new and relevant evidence. 2. *Modification of item 2 of the original GDC:* Decide who will be responsible for routinely monitoring the literature and assessing whether new significant evidence is available (e.g. consider involvement of experts not previously involved in the guideline development group to periodically review the guideline) and whether AI tools will be used to automatically monitor the literature.

^a^ Optimisation includes, as an example, model fine‐tuning or development of agentic AI models.

The results of user testing of the checklist extension to the ARIA 2024–2025 guidelines (Phase 4) are displayed in Supplementary Table 3. Overall, among steps that have been completed (i.e., all except those on “Dissemination and Implementation”, “Evaluation and Use” and “Updating”), we observed that there were 14 items that had been fulfilled, 11 items that had not been fulfilled, and 18 items which were not applicable/not performed (e.g., they concerned tasks in guideline development in which AI was not applied). The user testing resulted in minor suggestions of changes in the wording of four items (Supplementary Table 4).

## 4 Discussion

We have developed an extension for the GIN‐McMaster Guideline Development Checklist, specifically concerning the integration of AI in the health guideline enterprise. This extension was developed through an iterative process, involving both the use of AI tools and the input from experts.

The incorporation of AI‐tools in the health guideline enterprise can have several advantages, including–among others–the possibility of automating time‐consuming and repetitive tasks, streamlining processes, and the tailoring of materials to specific audiences. Overall, if adequately used, these tools can result in increasing efficiency and time gains. However, for such to occur, an initial upfront time and resources investment may be required, particularly in aspects such as training the elements of a guideline panel on the critical appraisal of AI‐based outputs, identification of the processes which may benefit the most from the use of AI and selection of the AI‐based tools/models to be used. Without such an investment, it may occur that AI can actually result in lower efficiency or in the generation of inadequate outputs. In fact, one of the GIN Principles for the use of AI in the health guideline enterprise–additionality–states that AI should bring gains in comparison with the sole use of non‐AI tools, namely through (i) performance of tasks that would not have been feasible by humans alone with the available resources, (ii) provision of complementary inputs to those provided by humans, and (iii) increase in the efficiency of tasks [4]. An inadequate preplanning and implementation of the use of AI can threaten the principle of additionality. By detailing all the different steps in which AI can be used in the health guideline enterprise, this checklist extension not only aims to enhance transparency but also to induce guideline panels in systematically discussing on how to maximise the potential of AI in terms of efficiency and quality gains.

The existence of a structured checklist extension can also help raising awareness to the limitations of an indiscriminate use of AI, including in the context of tasks without human validation or for which guideline panel members have no capacity to critically appraise the resulting outputs. This would not only threaten the principle of accountability (“humans should have oversight and direct the use of AI in the guideline enterprise so that they are accountable for the use of AI outputs and make sure that the use of AI is in line with current legal frameworks”) but could also pose concerns in terms of transparency [4]. The importance of human appraisal of AI‐generated content is reinforced by the results of the process followed to generate this checklist extension: less than 10% of the items generated by AI were integrated in the checklist extension without major modification. While the performance of AI can vary according to the task, it is essential to be able to critically appraise its outputs.

A comprehensive mapping on how this checklist extension can support the implementation of the GIN Principles for the use of AI in the health guideline enterprise is available in Supplementary Table 5.

### 4.1 Strengths and Limitations

This work has some strengths. From a methodological point of view, the development of this checklist extension was innovative, as it incorporated AI‐generated outputs. In fact, using ChatGPT 4.0 to propose a set of items which would be the starting point for further iterations was valuable experience on how AI‐generated insights can be incorporated into the guideline development process and on the limitations of taking the outputs at face value. In particular, most of the items proposed by ChatGPT 4.0 were not integrated into the final checklist extension, as they were redundant (i.e., often the same concept was suggested several times) or irrelevant. In particular, while some of the proposed items were (correctly) related to the integration of AI for guideline development, there were others which concerned the development of guidelines for integrating AI in healthcare. On the other hand, there were important omissions that were overcome by discussion among members of the working group.

An additional strength concerns the fact that the developed checklist was applied to a sample guideline, in particular to the ARIA 2024.2025 guidelines. This piloting has pointed to the potential usefulness of the checklist extension for a comprehensive and transparent use of AI in the guideline enterprise. In particular, we observed that the ARIA 2024‐2025 guidelines, despite using AI in several of its tasks, did not consider aspects such as dealing with CoI related to it.

There are some limitations to be considered. In particular, we have used a single LLM (ChatGPT 4.0) to generate the initial set of checklist items. In addition, we have used a single prompt to interact with ChatGPT 4.0. While several items suggested by ChatGPT 4.0 were included in the final checklist extension, it is possible to hypothesise that the testing of multiple prompts and the interaction with several LLMs could have resulted in a wider set of relevant items (even though previous studies comparing different LLMs in evidence‐related tasks found GPT‐based LLMs to be the ones with the best performance (Marques‐Cruz M, et al. Under review)). On the other hand, processes of iterative prompt improvement or of LLM fine‐tuning could have resulted in a lower amount of AI‐generated irrelevant results.

Another limitation is that, even though this checklist extension concerns the use of AI, several of its items are particularly applicable to the use of LLMs. This is justified by the fact that LLMs are probably the AI models most relevant in the context of the guideline enterprise. In fact, LLMs offer remarkable versatility in handling diverse language tasks like summarizing and producing text, translation and code adaptation, while being easily accessible through simple natural language interfaces [14]. However, it is important to note that AI is not restricted to LLMs.

Finally, for time‐related concerns, this checklist extension was not prospectively applied to any guideline. Instead, its application was retrospective and concerned a single guideline, having been done by a member of the steering group of the guidelines being assessed. An application of this checklist to multiple guidelines and/or by external independent reviewers would have resulted in a more robust evaluation, increasing the chance of identifying (i) relevant aspects that have not been covered, or (ii) items that would benefit from rewording. In any case, the ARIA 2024‐2025 guidelines have the advantage of having used AI in a diverse set of tasks, something that distinguishes them from most other guidelines.

### 4.2 Implications for Practice

This checklist extension is expected to be used throughout the whole health guideline enterprise process by the different involved stakeholders. It has been developed considering that the use of AI in the guideline process is becoming increasingly common, prompting a need for tools that help guideline developers complying with the principles for adequate use of AI in the guideline enterprise. We have developed this checklist extension without referring to specific tools or models, so that the adequacy of its items is expected to hold regardless of the AI tools being used or even of short‐term future advances in the AI field.

### 4.3 Implications for Research

Considering that this checklist extension has only been retrospectively applied, future studies may focus on its prospective application by external independent reviewers and in multiple guidelines. In particular, it would be desirable to quantify the time required for the use of this extension, as well as to evaluate its impact. Performing qualitative studies involving external teams who have prospectively applied this checklist extension may therefore help in its improvement.

Future research projects may also focus on testing multiple large language models in order to evaluate whether additional relevant items are proposed. If so, future research could focus on the development of a structured methodological framework to use AI‐based tools in (i) the appraisal of checklists and (ii) the support of creating checklist extensions.

Finally, and considering the rapid evolution of AI, regular meetings of the GIN group involved in the development of this checklist extension (with a periodicity of at least once per year) will be held to discuss the need for updating this extension.

## 5 Conclusion

We have developed an AI‐focused extension of the GIN‐McMaster Guideline Development Checklist. The extension items outline a minimum set of conduct standards that are intended to facilitate the comprehensive integration of AI tools throughout the health guideline enterprise. This tool has been developed as a result of the increasing use of AI in the health guideline process. Its prospective application may provide insights that can result in its further refinement and improvement.

## Author Contributions

**Bernardo Sousa‐Pinto:** conceptualization, writing – original draft, methodology. **Ignacio Neumann:** conceptualization, methodology, writing – original draft. **Yuan Chi:** conceptualization, writing – original draft, methodology. **Artur Nowak:** conceptualization, methodology, writing – original draft, writing – review and editing. **Marge Reinap:** methodology, writing – original draft, writing – review and editing, conceptualization. **Mariette Awad:** conceptualization, methodology, writing – original draft, writing – review and editing. **Amir Qaseem:** conceptualization, methodology, writing – original draft, writing – review and editing. **Wojtek Wiercioch:** conceptualization, writing – original draft. **Jan Brozek:** conceptualization, writing – original draft, writing – review and editing. **Holger J. Schünemann:** conceptualization, writing – original draft, writing – review and editing, methodology.

## Funding

The authors have nothing to report.

## Ethics Statement

The authors have nothing to report.

## Consent

The authors have nothing to report.

## Conflicts of Interest

AN and JB are employed and shareholders of Evidence Prime.

## Permission to Reproduce Material From Other Sources

Not applicable. This study does not reproduce material from other sources.

## Supporting information

Supporting File

## Data Availability

The authors have nothing to report.
