# Supplementary material for: GIN‐McMaster Guideline Development Checklist Extension for Integrating Artificial Intelligence in the Health Guideline Enterprise
Source: Clin Public Health Guidel. 2026 May 30;3(3):e70073. doi: 10.1002/gin2.70073 (PMC13563358; doi:10.1002/gin2.70073)
Supplement: Supplementary file 1 [file GIN2-3-e70073-s001.docx]

**Supplementary Tables**

Supplementary Table 1. Background information of the guideline developers who participated in the creation of the checklist extension

| **Name** | **Country** | **Gender** | **Occupation** | **Role in the project** |
| --- | --- | --- | --- | --- |
| Manuel Marques-Cruz | Portugal | Male | Data scientist | Initial item generation and review of AI outputs; manuscript writing |
| Bernardo Sousa-Pinto | Portugal | Male | Methodologist | Initial item generation and review of AI outputs; retrospective application of the checklist extension; manuscript writing |
| Ignacio Neumann | Chile | Male | Methodologist | Iterative refinement of the items; manuscript writing |
| Yuan Chi | China | Female | Data scientist | Scoping review planning; iterative refinement of the items; manuscript writing |
| Monika Nothacker | Germany | Female | Methodologist | Iterative refinement of the items; manuscript writing |
| Artur Nowak | Poland | Male | Software engineer | Iterative refinement of the items; manuscript writing |
| Marge Reinap | Denmark | Female | Policy-maker | Iterative refinement of the items; manuscript writing |
| Mariette Awad | Lebanon | Female | Computer engineer | Iterative refinement of the items; manuscript writing |
| Pablo Alonso-Coello | Spain | Male | Methodologist | Iterative refinement of the items; manuscript writing |
| Amir Qaseem | USA | Male | Methodologist | Iterative refinement of the items; manuscript writing |
| Elie A. Akl | Lebanon | Male | Methodologist | Methodology planning; iterative refinement of the items; manuscript writing |
| Wojtek Wiercioch | Canada | Male | Methodologist | Iterative refinement of the items; manuscript writing |
| Jan Brozek | Canada | Male | Methodologist | Iterative refinement of the items; manuscript writing |
| Holger J Schünemann | Italy | Male | Methodologist | Project coordination; methodology planning; iterative refinement of the items; manuscript writing |

Supplementary Table 2. Checklist extension items resulting from ChatGPT 4.0 prompting and iterative refinement.

| Topic | Additional Items for AI tools integration in guideline development |
| --- | --- |
| 1. Organization Budget Planning and Training | 1. Identify the specific AI tools that could be integrated into various stages of the guideline development process, such as evidence synthesis, data analysis, guideline dissemination, and implementation tracking. 2. Assess the technical and time requirements and infrastructure needed to support the integration of AI tools in the guideline development process, including software, hardware, and technical expertise. 3. Evaluate the costs associated with integrating AI tools into the guideline development process, including software licenses, training for team members, and potential ongoing support and maintenance fees. 4. Identify and establish partnerships with AI technology providers or academic institutions with expertise in AI to support the development and integration of these tools into the guideline development process. 5. *Correspondence with item 8 of the original GDC:* Plan for training sessions for guideline development team members on the use of AI tools, ensuring they understand how to effectively use these technologies in their work and that they understand their limitations. 6. Develop a framework to ensure AI-tools remain current throughout the guideline development process. |
| 2. Priority Setting | 1. Develop a framework for using AI for aiding in the collection of evidence on the different topics (e.g., on such aspects as their impact, recent evidence or availability of other recent guidelines) to support the priority setting process and guideline topic selection. 2. *Modification to item 9 of the original GDC:* Document the priority setting process, including how AI tools were used. |
| 3. Guideline Group Membership | 1. Include AI and data science experts in the guideline development group to provide expertise on the integration, application and limitations of AI tools within the guideline development process. 2. Consider the need for interdisciplinary collaboration, involving software developers, data analysts, and user experience designers, to develop and integrate AI tools effectively. 3. Establish roles for AI experts within the group, such as advising on AI tool selection, overseeing the development of custom AI solutions, and ensuring the quality and reliability of AI applications. |
| 4. Establishing Guideline Group Processes | 1. Establish a workflow for how AI-generated evidence and insights will be incorporated into the guideline development process, ensuring transparency and accountability in how AI outputs are used. 2. *Modification to item 3 of the original GDC:* As part of the training for the guideline development group, ensure that group members understand what the process and proposed methods will be and that they need to be adhered to (e.g. consensus methods that may be used, anonymous or non-anonymous voting, assessment of evidence and of AI-generated outputs, group discussion and contributing ideas). 3. Establish communication channels for collaboration between AI experts and other members of the guideline development group, to facilitate knowledge exchange and integration of AI into the guideline process. 4. *Adding to item 5 of the original GDC:* Determine how disagreements or conflicts regarding AI-based outputs or their interpretations will be resolved, ensuring a fair and evidence-based approach. 5. Implement feedback mechanisms to evaluate and continuously improve the impact of AI tools on the guideline development process, ensuring ongoing transparency and the ability to adapt to technological advances. |
| 5. Identifying Target Audience and Topic Selection | None. |
| 6. Consumer and Stakeholder Involvement | None. |
| 7. Conflict of Interest (COI) Considerations | 1. Extend the declaration of interest policy to include potential conflicts specific to AI tool development, deployment, and evaluation, such as financial interests in AI technologies that can be used in guideline development. 2. Require disclosure of any intellectual property interests in AI technologies that could influence recommendations or the selection of AI tools for guideline development. 3. Implement a policy for managing COI related to AI, such as excluding individuals with significant conflicts from making decisions on the selection of AI tools, while allowing for their contribution in areas where their expertise is essential but their COI does not influence outcomes. 4. Disclose and publish any conflicts of interest related to AI technologies among guideline group members, including the nature of the conflict and how it was managed, to maintain transparency and trust in the guideline development process. 5. Regularly update COI declarations to reflect any new AI-related interests or partnerships that could affect the integrity of the guideline development process. 6. Explicitly and transparently disclose whether AI tools were employed for supporting the assessment or management of conflicts of interest, and detail their specific applications. |
| 8. (PICO) Question Generation | 1. Detail how AI tools were applied for supporting the identification of PICO questions based on infodemiology data or data in natural language from other sources. 2. Detail how AI tools were applied for directly generating PICO questions, including prompts used and roles the AI tools were asked to assume. |
| 9. Considering Importance of Outcomes and Interventions | 1. Detail how AI tools were applied for supporting the identification of outcomes based on infodemiology data or data in natural language from other sources. 2. Detail how AI tools were applied for direct identification of potentially relevant outcomes, including prompts used and the roles the AI tools were asked to assume. 3. Describe the AI-assisted outputs in processes of evidence collection, analysis, and synthesis for systematic reviews of values and preferences. 4. Indicate whether AI tools were used for the appraisal of the risk of bias and indirectness of the evidence in the evaluation of values and preferences. 5. If AI tools were used for eliciting values & preferences or utilities, thoroughly and transparently record all the process, acknowledging the risk of algorithm biases resulting from the use of AI tools (e.g., risk of underrepresentation of the perspectives of some patient groups or populations). |
| 10. Deciding what Evidence to Include and Searching for Evidence | 1. Establish a process for engaging with AI developers and researchers to use AI tools for evidence search, analysis and/or synthesis. 2. Document all the processes in which AI tools were used (e.g., record selection, data extraction, risk of bias assessment, identification of unpublished data) to obtain, analyse and synthesise evidence required for the guideline, ensuring transparency and reproducibility. |
| 11. Summarizing Evidence and Considering Additional Information | 1. Document how AI tools were used to summarize evidence including based on information from different sources. |
| 12. Judging Quality Strength or Certainty of a Body of Evidence | 1. Detail how AI tools were utilized for evaluating the certainty in the body of evidence, clearly indicating the domains which were and were not automatically assessed, and how AI-based classifications were validated. 2. Evaluate the consistency of AI tool performance in the assessment of the certainty of evidence across different outcomes and comparisons. |
| 13. Developing Recommendations and Determining their Strength | 1. Detail the methods by which AI tools were utilized to collect, synthesize, and compile evidence, generate text, and perform other tasks supporting the development of recommendations, clearly delineating between AI-driven and human-driven roles in the development of recommendations.. 2. Select a method for assessing the quality and credibility of AI outputs in the context of the Evidence-to-Decision framework. |
| 14. Wording of Recommendations and Considerations | 1. Leverage AI tools to refine the drafting of recommendations, supporting a standardised (even if context-specific) wording with precise and unambiguous language. 2. Detail the application of AI tools for adaptation of recommendation wording to different stakeholders, including whether role-playing scenarios were employed to tailor recommendations for various stakeholders (e.g., patients of different backgrounds, healthcare providers, policymakers). |
| 15. Reporting and Peer Review | 1. Ensure the guideline report explicitly addresses how AI tools were used for guideline development, specifying the tools and methodologies employed. 2. Conduct a thorough review of the AI-specific outputs in the guideline by experts in AI, data science, and healthcare to ensure accuracy, relevance, and comprehensiveness. |
| 16. Dissemination and Implementation | 1. Create a detailed workflow that identifies areas where AI tools can enhance comprehensive dissemination strategies. This should include targeted and customized communication approaches for healthcare professionals, technology developers, and policymakers. 2. Detail how AI tools were used for the identification of obstacles and challenges to implementation of guideline recommendations. 3. Partner with healthcare technology organizations and AI developers to facilitate the integration of guideline recommendations into AI tools, ensuring alignment with best practices. |
| 17. Evaluation and Use | 1. Provide guidance on the criteria for modifying, continuing, or discontinuing the use of specific AI tools in the guideline development process based on evidence from evaluations and changing technological landscapes. 2. Detail if AI tools will be used to monitor and audit the implementation and effectiveness of guideline recommendations, including specific outcomes to track and methods for their assessment. |
| 18. Updating | 1. Secure resources and plan logistics for the efficient update of the guideline, considering the need for ongoing collaboration with AI developers and researchers to ensure that AI tools can be leveraged for identifying new and relevant evidence. |

Supplementary Table 3. Results of the user testing of the checklist extension to the Allergic Rhinitis and its Impact on Asthma (ARIA) 2024-2025 guidelines

| **Topic** | **Additional Items for AI tools integration in guideline development and description on their fulfilment in the ARIA 2024-2025 guidelines** | **Indication on whether the item was fulfiled** |
| --- | --- | --- |
| 1. Organization Budget Planning and Training | 1.       Identify the specific AI tools that could be integrated into specific stages of the guideline development process, such as evidence synthesis, data analysis, guideline dissemination, and implementation tracking.: *The use of specific AI tools that could be integrated into the different stages of guideline development has been described in the articles presenting the main principles^11^ and methodological approaches (Under review) of the ARIA 2024-2025 guidelines.* | Yes |
|  | 2.       Assess the technical and time requirements and infrastructure needed to support the integration of AI tools in the guideline development process, including software, hardware, and technical expertise.: *Not performed within the context of the ARIA guidelines* | No |
|  | 3.       Develop a framework to ensure that selected AI-tools will remain current throughout the guideline development process.: *Not performed within the context of the ARIA guidelines* | No |
|  | 4.       Evaluate the costs associated with integrating AI tools into the guideline development process, including software licenses, training for team members, and potential ongoing support and maintenance fees.: *Costs associated with integrating AI tools into the guideline development process were not evaluated at the planning stage of the ARIA guidelines* | No |
|  | 5.       Identify and establish partnerships with AI technology providers or academic institutions with expertise in AI to support the development and integration of these tools into the guideline development process.: *In the ARIA guidelines, we collaborated with academic and scientific institutions with expertise in AI, so that we could gather eventual support on how to best use them.* | Yes |
|  | 6.       Plan for training sessions for guideline development team members on the use of AI tools, ensuring they understand how to effectively use these technologies in their work and that they understand their limitations.: *The development of the ARIA guidelines did not require team members to use AI tools.* | Not applicable |
|  | 7.       Get  eventual needed approvals from advisory and/or regulatory bodies for the guideline development process.: *For the ARIA guidelines, there was no need for approval from advisory and/or regulatory bodies regarding the use of AI tools.* | Not applicable |
| 2. Priority Setting | 1.       Develop or apply a framework for using AI to support the priority setting process and guideline topic selection, for example to aid in the collection of evidence to inform priorities (e.g., assessing topic impact, identifying recent research evidence, assessing the availability and credibility of other recent guidelines, or identifying implementation issues and barriers to change).: *AI was not used to support the priority setting process or for guideline topic selection. The topic was the same of the previous ARIA guidelines.* | Not performed |
|  | 2.       Document the priority setting process, including how AI tools were used.: *AI tools were not used in the priority setting process.* | Not performed |
| 3. Guideline Group Membership | 1.       Include AI and data science experts in the guideline development group to provide expertise on the integration, application and limitations of AI tools within the guideline development process.: *AI and data science experts were included in the guideline development group.* | Yes |
|  | 2.       Consider the need for interdisciplinary collaboration, involving software developers, data analysts, and user experience designers, to develop and integrate AI tools effectively.: *The use of AI in the ARIA guidelines involved interdisciplinary collaboration between experts in AI, data science, research methodology and allergy. While we did not develop AI tools* de novo*, we developed an AI-based framework for complementing rapid evidence searches, with such development involving an interdisciplinary collaboration.* | Yes |
|  | 3.       Establish roles for AI experts within the group, such as advising on AI tool selection, overseeing the development of custom AI solutions, ensuring the quality and reliability of AI applications, and explaining AI-generated outputs and their limitations.: *Formal roles have not been defined for AI experts collaborating on the ARIA guidelines.* | No |
| 4. Establishing Guideline Group Processes | 1.       Establish a workflow for how AI-generated evidence and insights will be incorporated into the guideline development process, ensuring transparency and accountability in how AI outputs are used.: *In the ARIA guidelines, AI-generated evidence and insights was not used. However, we considered the output of tasks supported by AI, and for that a workflow was established on how such output would be incorporated into the guideline development process.* | Yes |
|  | 2.       As part of the training for the guideline development group, ensure that group members understand what the process and proposed methods will be and that they need to be adhered to (e.g. consensus methods that may be used, anonymous or non-anonymous voting, assessment of evidence and of AI-generated outputs, group discussion and contributing ideas).: *In the ARIA guidelines, we presented to the members of the guideline development group what were the processes in which AI was being used.* | Yes |
|  | 3.       Establish communication channels for collaboration between AI experts and other members of the guideline development group, to facilitate knowledge exchange and integration of AI into the guideline process.: *In the ARIA guidelines, the contacts of AI experts were made available to other members of the guideline development group.* | Yes |
|  | 4.       Determine how disagreements or conflicts regarding AI-based outputs or their interpretations will be resolved, ensuring a fair and evidence-based approach.: *There was no previous definition on how disagreements or conflicts regarding AI-based outputs or their interpretations would be resolved.* | No |
|  | 5.       Implement feedback mechanisms to evaluate and continuously improve the impact of AI tools on the guideline development process, ensuring ongoing transparency and the ability to adapt to technological advances.: *Feedback mechanisms were not implemented to evaluate and continuously improve the impact of AI tools on the guideline development process.* | No |
| 6. Consumer and Stakeholder Involvement | 1. Consider the use of AI tools to analyse or summarise the inputs of different stakeholders.: *AI tools were not used to summarise the inputs of different stakeholders.* | Not performed |
| 7. Conflict of Interest (COI) Considerations | 1.       Extend the declaration of interest policy to include potential conflicts specific to AI tool development, deployment, and evaluation, such as financial interests in AI technologies that can be used in guideline development.: *In the ARIA guidelines, conflicts specific to AI tool development, deployment, and evaluation were not assessed.* | No |
|  | 2.       Require disclosure of any intellectual property interests in AI technologies that could influence recommendations or the selection of AI tools for guideline development.: *Property interests in AI technologies were not evaluated.* | No |
|  | 3.       Implement a policy for managing COI related to AI, such as excluding individuals with significant conflicts from making decisions on the selection of AI tools, while allowing for their contribution in areas where their expertise is essential but their COI does not influence outcomes.: *A policy for managing conflicts of interest related to AI was not implemented.* | No |
|  | 4.       Disclose and publish any conflicts of interest related to AI technologies among guideline group members, including the nature of the conflict and how it was managed, to maintain transparency and trust in the guideline development process.: *Conflicts of interest related to AI technologies among guideline group members were not evaluated.* | No |
|  | 5.       Regularly update COI declarations to reflect any new AI-related interests or partnerships that could affect the integrity of the guideline development process.: *Updates of conflicts of interest declarations did not evaluate aspects on AI-related interests.* | No |
|  | 6.       Explicitly and transparently disclose whether AI tools were employed for supporting the assessment or management of conflicts of interest, and detail their specific applications.: *AI tools were not employed to support the assessment or management of conflicts of interest.* | Not performed |
| 8. (PICO) Question Generation | 1.       Detail how AI tools were applied for supporting the identification of PICO questions based on infodemiology data or data in natural language from other sources.: *The ARIA guidelines used AI tools to support the identification of PICO questions based on infodemiology data. Details are provided in a specific paper^13^.* | Yes |
|  | 2.       Detail how AI tools were applied for directly generating PICO questions, including prompts used and roles the AI tools were asked to assume.: *The ARIA guidelines used AI tools to directly generate PICO questions based on infodemiology data. Details are provided in a specific paper^13^.* | Yes |
| 9. Considering Importance of Outcomes and Interventions | 1.       Consider, plan and detail the application of AI tools for supporting the identification of health outcomes based on infodemiology data or data in natural language from other sources.*: The ARIA guidelines did not use AI tools to support the identification of health outcomes based on infodemiology data.* | Not performed |
|  | 2.       Consider, plan and detail the application of AI tools for direct identification of potentially relevant outcomes, including prompts used and the roles the AI tools were asked to assume.: *The ARIA guidelines used AI tools for direct generation of potentially relevant outcomes. Such process is described in an article describing the methodology of the ARIA guidelines (Under review)* | Yes |
|  | 3.       Describe the AI-assisted outputs in processes of evidence collection, analysis, and synthesis for systematic reviews of patients' values.: *The ARIA guidelines used an AI-assisted tool to support study selection in a systematic review of patients' values. The systematic review is described in a specific paper^14^.* | Yes |
|  | 4.       Indicate whether AI tools were used for the appraisal of the risk of bias and certainty of the evidence in the evaluation of patients’ values.: *AI tools were not used for the appraisal of the risk of bias and certainty of evidence in the evaluation of patients' values.* | Not performed |
|  | 5.       If AI tools were used for eliciting values and preferences or health utilities, thoroughly and transparently record the process, including considerations about the risk of algorithm biases resulting from the use of AI tools (e.g., risk of underrepresentation of the perspectives of some patient groups or populations ).: *In the ARIA guidelines, AI tools were not used for eliciting values and preferences or health utilities.* | Not performed |
| 10. Deciding what Evidence to Include and Searching for Evidence | 1.       Establish a process for engaging with AI developers and researchers to select and use AI tools for evidence search, analysis, and/or synthesis in systematic reviews that will be conducted.: *In the ARIA guidelines, AI tools were used for evidence search to complement rapid evidence reviews for guideline questions for which systematic reviews were not conducted. The framework used for AI use for evidence search is currently under review and experts were consulted to develop that framework.* | Yes |
|  | 2.       Document all the processes in which AI tools were used (e.g., record selection, data extraction, risk of bias assessment, identification of unpublished data) to obtain, analyse and synthesise evidence required for the guideline, ensuring transparency and reproducibility.: *In the ARIA guidelines, AI tools were used to search for evidence search to complement rapid evidence reviews for guideline questions for which systematic reviews were not conducted. The framework used for AI use for evidence search is currently under review. Information on the use of AI is described on the corresponding guidelines paper (under review).* | Yes |
| 11. Summarizing Evidence and Considering Additional Information | 1.       Document how AI tools were used to summarize evidence including based on information from different sources (e.g., systematic reviews, collection of primary data, etc.).: *AI tools were not used to summarise evidence based on information from different sources.* | Not performed |
| 12. Judging Quality Strength or Certainty of a Body of Evidence | 1.       Decide who will be responsible for appraising the quality of evidence (e.g. un-conflicted methodologists participating in the working group) and whether AI tools will be used as a support.: *Automated tools were used to support the assessment of the certainty of evidence in the context of some network meta-analyses (automated supported application of GRADE NMA). However, AI was not used in those automated tools (only predefined rules).* | Not performed |
|  | 2.       Document how AI tools were used for evaluating the certainty in the body of evidence, clearly indicating the domains which were and were not assessed with AI assistance, and how AI-based classifications were validated.: *AI tools were not used for evaluating the certainty in the body of evidence. However, we used non-AI-based automated tools to support the assessment of the certainty of evidence in the context of some network meta-analysis. Such use has been described in the corresponding guideline paper (guideline on oral interventions for allergic rhinitis).* | Not performed |
|  | 3.       Ensure the consistency of AI tool performance in the assessment of the certainty of evidence across different outcomes and comparisons in the guideline.: *AI tools were not used for evaluating the certainty in the body of evidence. However, we used non-AI-based automated tools to support the assessment of the certainty of evidence in the context of some network meta-analysis. Since these tools are based on predefined rules, output inconsistency is not expected.* | Not performed |
| 13. Developing Recommendations and Determining their Strength | 1.       Document the methods by which AI tools were used to collect, synthesize, and compile evidence, generate text, and perform other tasks supporting the development of recommendations, clearly delineating between AI-driven and human-driven roles in the development of recommendations.: *AI tools were not used for making judgements or developing recommendations in the context of the ARIA guidelines.* | Not performed |
|  | 2.       Select a method for assessing the quality and credibility of AI outputs in the context of the Evidence-to-Decision framework.: *AI tools were not used for making judgements or developing recommendations in the context of the ARIA guidelines.* | Not performed |
| 14. Wording of Recommendations and Considerations of Implementation, Feasibility and Equity | 1.       Document the use of AI tools in the drafting of recommendation wording (e.g., generative wording in a standardised format with precise and unambiguous language).: *AI tools were not used for drafting recommendations in the context of the ARIA guidelines.* | Not performed |
|  | 2.       Document the use of AI tools for adaptation of recommendation wording to different stakeholders, including whether role-playing scenarios were employed to tailor recommendations for various stakeholders (e.g., patients of different backgrounds, healthcare providers, policymakers).: *AI tools were not used for adapting the wording of recommendations in the context of the ARIA guidelines.* | Not performed |
| 15. Reporting and Peer Review | 1.       Ensure the guideline report explicitly addresses how AI tools were used for guideline development, specifying the tools and methodologies employed.: *The guideline reports explicitly address how AI tools were used for guideline developent (^11^ and manuscripts under review).* | Yes |
|  | 2.       Conduct a thorough review of the AI-specific outputs in the guideline by experts in AI, data science, and healthcare to ensure accuracy, relevance, and comprehensiveness.: *Not fully applicable, since AI-based outputs in the ARIA guidelines concerned question generation, outcome generation and evidence search (as a complement to rapid evidence reviews)* | Not applicable |
| 16. Dissemination and Implementation | 1.       Create a detailed workflow that identifies areas where AI tools can enhance comprehensive dissemination strategies. This should include targeted and customized communication approaches for healthcare professionals, technology developers, and policymakers.: *In the conceptualization and description of the methods of the ARIA guidelines, dissemination and implementation aspects that would benefit from the use of AI (production of plain language summaries tailored to different audiences; integration of recommendations in digital care pathways in MASK-air) have been identified.* | Yes |
|  | 2.       Document how AI tools were used for creation of dissemination and implementation products (e.g., writing of plain language summaries, design of decision-aids and guideline summaries).: *To be performed once ARIA guidelines are completed* | To be  performed |
|  | 3.       Detail how AI tools were used for the identification and support of the overcome of obstacles and challenges to implementation of guideline recommendations.: *Not planned to be performed in the context of the ARIA guidelines.* | Not to be  performed |
|  | 4.       Partner with healthcare technology organizations and AI developers to facilitate the integration of guideline recommendations into AI tools, ensuring alignment with best practices.: *To be performed once ARIA guidelines are completed, with integration of their recommendations in digital care pathways to be embedded in the MASK-air app.* | To be  performed |
| 17. Evaluation and Use | 1.       Provide guidance on the criteria for modifying, continuing, or discontinuing the use of specific AI tools in the guideline development process based on evidence from evaluations and changing technological landscapes.: *To be performed once ARIA guidelines are complete.* | To be  performed |
|  | 2.       Detail if AI tools will be used to monitor and audit the implementation and effectiveness of guideline recommendations, including specific outcomes to track and methods for their assessment.: *AI tools are not planned to be used to monitor and audit the implementation and effectiveness of guideline recommendations.* | Not to be  performed |
| 18. Updating | 1.       Secure resources and plan logistics for the efficient update of the guideline, considering the need for ongoing collaboration with AI developers and researchers to ensure that AI tools can be leveraged for identifying new and relevant evidence.: *Not yet performed.* | Not performed |
|  | 2.       Decide who will be responsible for routinely monitoring the literature and assessing whether new significant evidence is available (e.g. consider involvement of experts not previously involved in the guideline development group to periodically review the guideline) and whether AI tools will be used to automatically monitor the literature.: *Not yet performed.* | Not performed |

Supplementary Table 4. Suggestions of changes in the wording of items after the user testing of the checklist extension

| **Topic** | **Original item** | **Suggested modified item** |
| --- | --- | --- |
| 3. Guideline Group Membership | 2.       Consider the need for interdisciplinary collaboration, involving software developers, data analysts, and user experience designers, to develop and integrate AI tools effectively | 2.       Consider the need for interdisciplinary collaboration, involving software developers, data analysts, and user experience designers, to develop, integrate and use AI tools effectively |
| 4. Establishing Guideline Group Processes | 1.       Establish a workflow for how AI-generated evidence and insights will be incorporated into the guideline development process, ensuring transparency and accountability in how AI outputs are used | 1.       Establish a workflow for how AI-generated evidence, insights and related outputs will be incorporated into the guideline development process, ensuring transparency and accountability in how AI outputs are used |
| 13. Developing Recommendations and Determining their Strength | 1.       Document the methods by which AI tools were used to collect, synthesize, and compile evidence, generate text, and perform other tasks supporting the development of recommendations, clearly delineating between AI-driven and human-driven roles in the development of recommendations | 1.       Document the methods by which AI tools were used for tasks supporting the development of recommendations, clearly delineating between AI-driven and human-driven roles in the development of recommendations |
| 13. Developing Recommendations and Determining their Strength | 2.       Select a method for assessing the quality and credibility of AI outputs in the context of the Evidence-to-Decision framework | 2. Select a method for assessing the quality and credibility of AI outputs for supporting judgements and recommendations in the context of the Evidence-to-Decision framework |

Supplementary Table 5. Description on how the checklist extension supports the implementation of each Guidelines International Network principle for use of artificial intelligence (AI) in the health guideline enterprise.

| **Principle** | **Description on how the checklist extension supports the implementation of the principle** |
| --- | --- |
| **Transparency** | The checklist extension will support the implementation of the transparency principle, by making sure that the use of AI throughout all stages of the guideline enterprise will be adequately documented. The following items are particularly related with the implementation of the transparency principle:   1. Document the priority setting process, including how AI tools were used. 2. Explicitly and transparently disclose whether AI tools were employed for supporting the assessment or management of conflicts of interest, and detail their specific applications. 3. Detail how AI tools were applied for supporting the identification of PICO questions based on infodemiology data or other sources of data. 4. Detail how AI tools were applied for directly generating PICO questions, including prompts used, the roles the AI tools were asked to assume, and whether there were was an evaluation of the potential impact of AI biases in the direct generation of questions. 5. Document all the processes in which AI tools were used (e.g., record selection, data extraction, risk of bias assessment, identification of unpublished data) to obtain, analyse and synthesise evidence required for the guideline, ensuring transparency and – as much as possible - reproducibility. 6. Document how AI tools were used to extract data and summarize evidence including based on information from different sources (e.g., systematic reviews, collection of primary data, etc.). Detail the human validation process for AI-extracted data and summaries, including error rates or agreement metrics if assessed. 7. Describe the AI-assisted outputs in processes of evidence collection, analysis, and synthesis for systematic reviews of patients' values. 8. Document how AI tools were used for evaluating the certainty in the body of evidence, clearly indicating the domains which were and were not assessed with AI assistance, and how AI-based classifications were validated. 9. Document the methods by which AI tools were used to support the development of recommendations, clearly delineating between AI and human roles in the development of recommendations. 10. Document the use of AI tools in the drafting of recommendation wording (e.g., generative wording in a standardised format with precise and unambiguous language). 11. Document the use of AI tools for adaptation of recommendation wording to different stakeholders, including whether role-playing scenarios were employed to tailor recommendations for various stakeholders (e.g., patients of different backgrounds, healthcare providers, policymakers). 12. Ensure the guideline report explicitly addresses how AI tools were used for guideline development, specifying the tools and methodologies employed. 13. Document how AI tools were used for creation of dissemination and implementation products (e.g., writing of plain language summaries, design of decision-aids and guideline summaries). 14. Detail how AI tools were used for the identification and support of the overcome of obstacles and challenges to implementation of guideline recommendations. 15. Detail if AI tools will be used to monitor and audit the implementation and effectiveness of guideline recommendations, including specific outcomes to track and methods for their assessment. |
| **Preplanning** | The checklist extension includes several items that aim to make sure that there is an adequate planning of the use of AI tools in the different stages of the guideline enterprise. Such items concern aspects such as technical resources, time requirements and costs:   7. Establish a workflow for how AI-generated evidence, insights and related outputs will be incorporated into the guideline development process, ensuring transparency and accountability in how AI outputs are used. 8. Identify the specific AI tools that could be integrated into specific stages of the guideline development process, such as evidence synthesis, data analysis, guideline dissemination, and implementation tracking. 9. Assess the technical and time requirements and infrastructure needed to support the integration of AI tools in the guideline development process, including software, hardware, and technical expertise. 10. Evaluate the costs associated with integrating AI tools into the guideline development process, including software licenses, training for team members, and potential ongoing support and maintenance fees. 11. Secure resources and plan logistics for the efficient update of the guideline, considering the need for ongoing collaboration with AI developers and researchers to ensure that AI tools can be leveraged for identifying new and relevant evidence. |
| **Additionality** | The principle of “additionality” posits that AI should bring gains in comparison with the sole use of non-AI tools. While this principle is transversal to the whole guideline enterprise, this checklist extension suggests some processes in which the use of AI could be of particular benefit:   1. Consider the use of AI tools to analyse or summarise the inputs of different stakeholders. 2. Consider, plan and detail the application of AI tools for supporting the identification of health outcomes based on infodemiology data or data in natural language from other sources. 3. Consider, plan and detail the application of AI tools for direct identification of potentially relevant outcomes, including prompts used and the roles the AI tools were asked to assume. 4. Create a detailed workflow that identifies areas where AI tools can enhance comprehensive dissemination strategies. This should include targeted and customized communication approaches for healthcare professionals, technology developers, policymakers, patients and the public. 5. Partner with healthcare technology organizations and AI developers to facilitate the integration of guideline recommendations into AI tools (e.g., AI-based clinical decision support systems or interactive bots), ensuring alignment with best practices. |
| **Credibility** | The checklist extension supports the implementation of the credibility principle by having items that address the need for evaluating the performance/quality of AI tools in the corresponding tasks:   1. Thoroughly and transparently record the processes involving the use of AI tools for eliciting values and preferences or health utilities, including considerations about the risk of algorithm biases resulting from the use of AI tools (e.g., risk of underrepresentation of the perspectives of some patient groups or populations). 2. Establish a process for engaging with AI developers and researchers to select and use AI tools for evidence search, analysis, and/or synthesis in systematic reviews that will be conducted. 3. Ensure the consistency of AI tool performance in the assessment of the certainty of evidence across different outcomes and comparisons in the guideline. 4. Define methods for assessing the quality and credibility of AI outputs for supporting judgements and recommendations in the context of the Evidence-to-Decision framework. |
| **Ethics** | While all the items of the checklist presuppose an ethical use of AI, there are some which specifically deal with managing conflicts of interest in relation to the use of AI:   1. Extend the declaration of interest policy to include potential conflicts specific to AI tool development, deployment, and evaluation, such as financial interests in AI technologies that can be used in guideline development. 2. Require disclosure of any intellectual property interests in AI technologies that could influence recommendations or the selection of AI tools for guideline development. 3. Implement a policy for managing COI related to AI, such as excluding individuals with significant conflicts from making decisions on the selection of AI tools, while allowing for their contribution in areas where their expertise is essential but their COI does not influence outcomes. 4. Disclose and publish any conflicts of interest related to AI technologies among guideline group members, including the nature of the conflict and how it was managed, to maintain transparency and trust in the guideline development process. 5. Regularly update COI declarations to reflect any new AI-related interests or partnerships that could affect the integrity of the guideline development process. |
| **Accountability** | The accountability principle posits that humans should have oversight and direct the use of AI in the guideline enterprise. The checklist extension supports the implementation of this principles by having items (i) dedicated to the participation of AI and data science experts in the guideline enterprise, (ii) ensuring that members of the guideline development team are trained on the use of AI and/or interpretation of its outputs, and that (iii) roles in specific tasks involving the use of AI are clearly defined:   1. Identify and establish partnerships with AI technology providers or academic institutions with expertise in AI to support the optimisation and/or integration of these tools into the guideline development process. 2. Plan for training sessions for guideline development team members on the use of AI tools, ensuring they understand how to effectively use these technologies in their work and that they understand their limitations. 3. Include AI and data science experts in the guideline development group to provide expertise on the integration, application and limitations of AI tools within the guideline development process. 4. Consider the need for interdisciplinary collaboration, involving software developers, data scientists/analysts, among others, to develop/optimise, integrate and use AI tools effectively. 5. Establish roles for AI experts within the group, such as advising on AI tool selection, overseeing the development of custom AI solutions, ensuring the quality and reliability of AI applications, and explaining AI-generated outputs and their limitations. 6. Establish communication channels for collaboration between AI experts and other members of the guideline development group, to facilitate knowledge exchange and integration of AI into the guideline process. 7. Decide who will be responsible for appraising the quality of evidence (e.g. un-conflicted methodologists participating in the working group) and whether AI tools will be used as a support. 8. Conduct a thorough review of the AI-specific outputs in the guideline by experts in AI, data science, and healthcare to ensure accuracy, relevance, and completeness. 9. Decide who will be responsible for routinely monitoring the literature and assessing whether new significant evidence is available (e.g. consider involvement of experts not previously involved in the guideline development group to periodically review the guideline) and whether AI tools will be used to automatically monitor the literature. |
| **Compliance** | The checklist extension presupposes that the use of AI in the guideline enterprise must comply with legal and regulatory frameworks. This is stated in the following item:   1. Get eventual needed approvals from advisory and/or regulatory bodies for the guideline development process. |
| **Evaluation** | The checklist extension will support the evaluation principle by ensuring monitoring and assessment of the use of AI throughout the different stages of the guideline enterprise:   1. Develop a framework to ensure that selected AI-tools will remain current throughout the guideline development process. 2. Implement feedback mechanisms to evaluate and continuously improve the impact of AI tools on the guideline development process, ensuring ongoing transparency and the ability to adapt to technological advances. 3. Provide guidance on the criteria for modifying, continuing, or discontinuing the use of specific AI tools in the guideline development process based on evidence from evaluations and changing technological landscapes. For example, after guideline completion, evaluate the efficiency, cost-effectiveness and quality impact of using AI tools in the development process. |

COI: Conflict of interest; PICO: Population Intervention Comparison Outcome
